# Single-Base Substitution Causing Dual-Exon Skipping Event in *PKD2* Gene: Unusual Molecular Finding from Exome Sequencing in a Patient with Autosomal Dominant Polycystic Kidney Disease

**DOI:** 10.3390/jcm13164682

**Published:** 2024-08-09

**Authors:** Elisa De Paolis, Giuseppina Raspaglio, Nunzia Ciferri, Ilaria Zangrilli, Claudio Ricciardi Tenore, Andrea Urbani, Pietro Manuel Ferraro, Angelo Minucci, Paola Concolino

**Affiliations:** 1 Departmental Unit of Molecular and Genomic Diagnostics, Fondazione Policlinico Universitario A. Gemelli IRCCS, 00168 Rome, Italy; elisa.depaolis@policlinicogemelli.it (E.D.P.); giuseppina.raspaglio@unicatt.it (G.R.); ilaria.zangrilli@policlinicogemelli.it (I.Z.); claudio.ricciarditenore@guest.policlinicogemelli.it (C.R.T.); angelo.minucci@policlinicogemelli.it (A.M.); 2 Clinical Chemistry, Biochemistry and Molecular Biology Operations (UOC), Fondazione Policlinico Universitario A. Gemelli IRCCS, 00168 Rome, Italy; andrea.urbani@policlinicogemelli.it; 3 Division of Oncological Gynecology, Department of Women’s and Children’s Health, Fondazione Policlinico Universitario A. Gemelli IRCCS, 00168 Rome, Italy; 4 Nephrology Unit, Departement of Medical and Surgical Sciences, Fondazione Policlinico Universitario A. Gemelli IRCCS, 00168 Rome, Italy; nunzia.ciferri01@icatt.it; 5 Department of Basic Biotechnological Sciences, Intensivological and Perioperative Clinics, Catholic University of Sacred Heart, 00168 Rome, Italy; 6 Section of Nephrology, Department of Medicine, Università degli Studi di Verona, 37127 Verona, Italy; pietromanuel.ferraro@univr.it

**Keywords:** autosomal dominant polycystic kidney disease, PKD2, splicing, next-generation sequencing, exome sequencing

## Abstract

**Background**: Pathogenic variants in the Polycystic Kidney Disease 2 (PKD2) gene are associated with Autosomal Dominant Polycystic Kidney Disease (ADPKD) in approximately 30% of cases. In recent years, the high-throughput sequencing techniques have significantly increased the number of variants identified in affected patients. Here, we described the peculiar effect of a *PKD2* splicing variant, the c.1717-2A>G, identified in an Italian male patient with ADPKD. This variant led to the unusual and rare skipping of two consecutive exons, causing a large in-frame deletion. **Methods**: The genetic evaluation of the patient was performed using the Next-Generation Sequencing (NGS) assay Clinical Exome Solution^®^ (SOPHiA Genetics). Bioinformatics analysis was performed using the SOPHiA DDM platform (SOPHiA Genetics). Prediction of pathogenicity was carried out by integrating several in silico tools. RNA evaluation was performed to test the effect of the variant on the *PKD2* splicing using a Reverse-Transcription PCR coupled with cDNA sequencing. **Results**: NGS revealed the presence of the *PKD2 c.1717-2A>G* variant that lies in the canonical splice site of intron 7. This rare variant was predicted to have a significant impact on the splicing, proved by the RNA-based analysis. We identified the presence of a transcript characterised by the simultaneous skipping of exons 8 and 9, with a retained reading frame and the merging of exons 7–10. **Conclusions**: We described for the first time a dual-exon skip event related to the presence of a single-base substitution in the *PKD2* gene in an ADPKD-affected patient. We assumed that the molecular basis of such a rare mechanism lies in the specific order of intron removal. The finding represents novel evidence of an alternative and unusual splicing mechanism in the *PKD2* gene, adding insights to the pathogenesis of the ADPKD.

## 1. Introduction

Autosomal Dominant Polycystic Kidney Disease (ADPKD) (OMIM# 173900) is considered the most prevalent monogenic nephropathy, with an estimated prevalence of 1:100–2500 individuals, and one of the most common inherited disorders [1,2,3]. ADPKD is characterised by the development and expansion of multiple cysts scattered throughout the kidney parenchyma. Over time, progressive loss of kidney function occurs, leading to end-stage kidney disease during or after the sixth decade of life. Most individuals with enlarged kidneys or decreased glomerular filtration rate (GFR) show hypertension, a very common complication in ADPKD. Chronic pain, gross haematuria, cyst infection, and nephrolithiasis are some of the complications that arise from cyst growth and expansion. In addition, affected individuals might exhibit extrarenal manifestations, including hepatic and pancreatic cysts, intracranial aneurysms, abdominal hernias, and cardiac valvular lesions. Finally, complications due to the decrease in GFR can over time cause anaemia, secondary hyperparathyroidism, metabolic bone disease, inadequate nutrition, and increased cardiovascular risk [4,5,6,7].

In 85% of cases where pathogenic genetic variants are found, ADPKD is the result of mutations in Polycystic Kidney Disease 1 (*PKD1*, OMIM #601313) (70% of cases) and Polycystic Kidney Disease 2 (*PKD2*, OMIM #613095) (30% of cases) genes [8,9]. Additionally, alterations of some other genes, such as *GANAB* (OMIM#104,160) and *DNAJB11* (OMIM#611,341), could have an impact on the folding, maturation, and transport of PKD1 and PKD2 protein products, contributing to the development of cysts [10,11,12]. *PKD1*, located on chromosome 16 (16p13.3), encodes for polycystin-1 (PC1), a large multidomain glycoprotein containing an N-terminal extracellular region, multiple transmembrane domains, and a cytoplasmic C-tail. PC1 is an integral membrane protein involved in calcium permeable cation channel regulation and intracellular calcium homeostasis. PC1 is also involved in cell–cell/matrix interactions and may modulate G-protein-coupled signal-transduction pathways [13,14]. *PKD2*, located on chromosome 4 (4q21), encodes polycystin-2 (PC2), a multi-pass membrane protein belonging to the transient receptor potential family of calcium-regulated cation channels. PC2 is involved in calcium transport and calcium signalling in renal epithelial cells [15]. PC1 forms a complex with PC2 that regulates multiple signalling pathways to maintain normal renal tubular structure and function [16]. Both these proteins are located on the primary cilia for the transmission of signalling from the external environment to the cell. Some evidence supports that PC1 and PC2 inhibit cystogenesis in a dose-dependent way, and that cystogenesis occurs when the concentration of PC1 or PC2 falls below a certain threshold [17,18].

Over recent years, the use of high-throughput sequencing techniques based on large-scale parallel sequencing has rapidly increased the number of identified variants in ADPKD-related genes. To date, more than 1400 different pathogenic and likely pathogenic variants of *PKD1* and *PKD2* genes have been indexed in the ADPKD mutation database (https://pkdb.mayo.edu/welcome, accessed on 15 June 2024). Approximately 300 disease-causing variants have been reported in the *PKD2* gene, where frameshift and nonsense variant types were the most identified. Several splicing variants have also been described as pathogenic or likely pathogenic (https://www.ncbi.nlm.nih.gov/clinvar, accessed on 15 June 2024).

In this study, we described an Italian patient affected by ADPKD carrying a peculiar splicing variant in the *PKD2* gene, the *c.1717-2A>G* identified through a clinical exome sequencing strategy. Our molecular evaluation indicated that this single-base substitution, lying in the canonical acceptor splice-site of intron 7, leads to the unusual skipping of the two consecutive exons 8 and 9, causing an in-frame deletion of 100 amino acids.

## 2. Materials and Methods

### 2.1. Patient

A 48-year-old Italian man came to the Kidney Outpatient Clinic of the Fondazione Policlinico Universitario “A. Gemelli” for the first time in February 2022. The clinical diagnosis of ADPKD was previously carried out at another medical centre, but no genetic analysis was offered although the proband’s mother and sister were also affected. The man had no intracranial aneurysms on magnetic resonance imaging angiography and no pancreatic cysts. He had some liver cysts on abdominal ultrasound and reported an episode of post-traumatic cyst haemorrhage, for which he underwent embolisation. No other medical conditions were referred other than hypertension. An abdomen computed tomography for the determination of kidney volumes revealed the following: right 1325 cc, left 1175 cc, and ht-TKV 1445 cc/m. On the biochemical tests, the following results were shown: serum creatinine 1.39 mg/dL (reference range: 0.67–1.20 mg/dL), blood urea nitrogen 21 mg/dL (reference range: 10–23 mg/dL), sodium 138 mmol/L (reference range: 135–145 mmol/L), potassium 3.8 mmol/L (reference range: 3.5–5.0 mmol/L), calcium 9.4 mg/dL (reference range: 8.7–10.3 mg/dL), estimated GFR (eGFR) 51 mL/min/1.73 m^2^ (Chronic Kidney Disease Epidemiology Collaboration (CKD-EPI) 2021 equation; reference cut-off ≥90 mL/min/1.73 m^2^), urine specific gravity 1.017 (reference range: 1.003–1.035), and proteinuria 150 mg/24 h (reference cut-off <150 mg/24 h). The patient was on therapy with perindopril, indapamide, and amlodipine, and he was introducing 1.5 L of water daily. We recommended him to discontinue indapamide to avoid the vasopressin-elevating effect of the diuretic, increase hydration to 3 L per day, reduce the intake of animal protein and salt, limit coffee to no more than 1 cup per day, and continue the home monitoring of blood pressure.

To achieve a genetic diagnosis of ADPKD, the patient and his affected relatives were addressed to the Departmental Unit of Molecular and Genomic Diagnostics of the Fondazione Policlinico Universitario “A. Gemelli” in April 2022 in order to perform the molecular investigation. All procedures were made in accordance with the standards of the Ethics Committee of Fondazione Policlinico Universitario “A. Gemelli” IRCCS of Rome. The patient signed written informed consent before the genetic test and received counselling before and after the results.

### 2.2. Molecular Analysis and In Silico Prediction

Genomic DNA was extracted starting from a peripheral blood draw with an automatic procedure performed using the QIAmp DNA Mini kit on the Qiacube platform (Qiagen, Milan, Italy) following the manufacturer’s indications. Quality checks of the extracted DNA were obtained using the Qubit dsDNA BR fluorometric assays (Life Technologies, Gaithersburg, MD, USA) for quantitation and the spectrophotometry NanoDrop^®^ ND-1000 (Life Technologies, Gaithersburg, MD, USA) for purity evaluation. We adopted a Next-Generation Sequencing (NGS) approach to analyse a panel of genes known to be associated with cystic kidney disease using the Clinical Exome Solution^®^v3 kit (CES, SOPHiA Genetics, Saint Sulpice, Switzerland).

The sequencing library was prepared starting from 100 ng of DNA. According to the manufacturer’s protocol, DNA fragments were generated using an enzymatic fragmentation step. The subsequent enzymatic steps (i.e., end-repair, A-tailing, and ligation to Illumina adapters) were performed in order to produce an NGS library. A capture-based target enrichment was carried out on pooled libraries. Quantitation of the final pool of libraries was performed using the Qubit dsDNA HS fluorimetric assays (Life Technologies, Gaithersburg, MD, USA). Quality control of fragment size was assessed using HS DNA ScreenTape analysis (TapeStation system, Agilent Technologies, Palo Alto, CA, USA).

The sequencing protocol was performed in 151 × 151 paired-end reads mode on the Illumina NextSeq550DX^®^ instrument (Illumina, San Diego, CA, USA) according to manufacturer’s multiplexing. Output FastQ files were analysed by the Sophia DDM^®^ platform (Sophia Genetics, Saint Sulpice, Switzerland) to evaluate single-nucleotide variants (SNVs), insertions and deletions (indels), and Copy Number Variants (CNVs). Bioinformatics prediction of CNVs was performed by analysing the coverage levels of the target regions across samples. Identified variants were interpreted in accordance with the American College of Medical Genetics (ACMG) guidelines [19] and the main mutational and population databases as ClinVar (https://www.ncbi.nlm.nih.gov/clinvar/, accessed on 15 June 2024), LOVD (https://databases.lovd.nl/shared/genes/PKD2, accessed on 15 June 2024), ADPKD Variant Database (https://pkdb.mayo.edu/welcome, accessed on 15 June 2024), and gnomADv4.0 (https://gnomad.broadinstitute.org/, accessed on 15 June 2024). The effect of the identified variant on the splicing process was assessed by using the MobyDetails tool that collects several in silico platforms to allow a complete evaluation of the splicing impairment [20]. Integrated prediction resulted in the following: MoBiDiC Prioritization Algorithm (MPA), score 0–10 (high impact: 10) [21]; MaxEntScan (∆MaxEnt Var − ∆MaxEnt WT < 0 for potential loss of donor/acceptor site) [22], dbscSNV ADA, and RF, score 0–1 (threshold ≥ 0.8 for impact) [23]; spliceAI Acceptor Gain (AG), Acceptor Loss (AL), Donor Gain (DG), Donor Loss (DL), score 0–1 (thresholds ≥ 0.2|0.5|0.8 for impact) [24].

### 2.3. cDNA Analysis

Total RNA was extracted from 5 mL of peripheral blood using the blood total RNA purification kit (Qiagen, Milan, Italy) following the manufacturer’s protocol. RNA concentration was determined using Qubit 2.0 with the RNA High Sensitivity kit (Life Technologies, Gaithersburg, MD, USA). Nucleic acid purity was assessed by Nanodrop (Life Technologies, Gaithersburg, MD, USA), evaluating the ratio of the absorbance at 260 nm/280 nm and 260 nm/230 nm. The RNA integrity number was assessed with the Agilent RNA ScreenTape kit on a TapeStation 4200 automated electrophoresis system (Agilent Technologies, Palo Alto, CA, USA). Double-strand cDNA was obtained from 100 ng of total RNA using the PreAmp and Reverse Transcription kit (Fluidigm, Standard Biotools, San Francisco, CA, USA). cDNA was then pre-amplified using oligonucleotides designed ad hoc for the amplification of the *PKD2* cDNA region encompassing exon 7 to the first 54 bp of exon 10. Primer sequences consisted of forward 5′-TGTGCTGTCAGTGGTAGCTA-3′ and reverse 5′-CTCTGAAGTGAAATCTGACTTG-3′ (NM_000297.4). Unincorporated primers were eliminated by an exonuclease I treatment at 37 °C for 30 min. In the final step, the pre-amplified and exonuclease I-treated cDNA was diluted 1:20 and amplified by PCR with the same primers used in the pre-amplification reaction. PCR reactions were evaluated on 2% of agarose gel and by means of an Agilent High Sensitivity DNA ScreenTape kit on a TapeStation 4200 automated electrophoresis system (Agilent Technologies, Palo Alto, CA, USA). According to the primer pair designs, the wild-type PCR product was expected to be 529 bp long while that of the supposed alternative fragment, resulting from the exon 8 skipping, was assumed to be of 344 bp.

The obtained PCR products were purified by gel extraction with the PCR clean-up gel extraction kit (Macherey-Nagel, Duren, Germany). Sequencing of the purified PCR products was performed using a BigDye Terminator Cycle Sequencing Kit v3.1 (Life Technologies, Gaithersburg, MD, USA) on the ABI 3500 Genetic Analyzer (Life Technologies, Gaithersburg, MD, USA). Results were analysed with the SeqScape v2.5 software package (Life Technologies, Gaithersburg, MD, USA) and the Chromas 2.5 tool (https://technelysium.com.au/wp/, accessed on 15 June 2024) using NM_000297.4 as the reference. The same cDNA analysis was performed using 10 blood samples of controls.

## 3. Results

### 3.1. Molecular Analysis and In Silico Prediction

**Figure 1:**
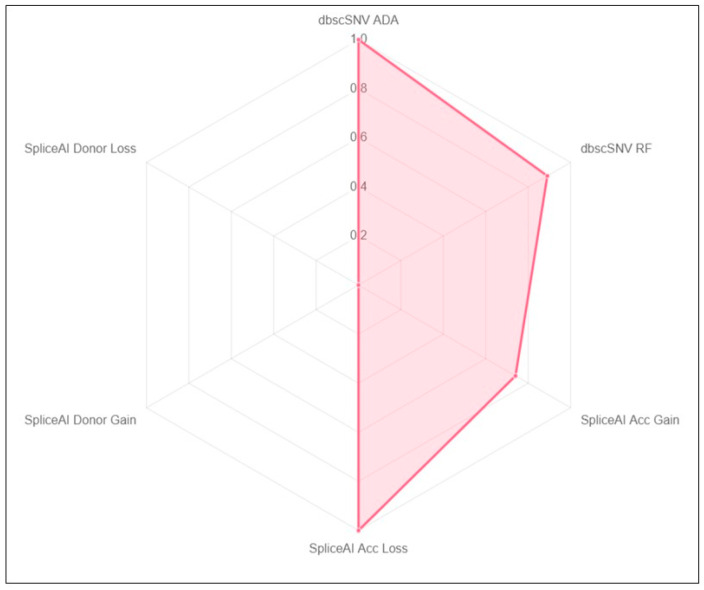
Splicing predictor outputs. This figure shows the radar view of splicing predictors obtained from Moby Details analysis (details in the text).

The molecular analysis was performed only on the proband, while his relatives declined the test. NGS identified the heterozygous splicing variant, *c.1717-2A>G,* in the *PKD2* gene. No additional variants or CNVs were detected in the analysed genes. The *PKD2 c.1717-2A>G* variant, involving the canonical splice site of intron 7, is a rare alteration not currently annotated in the main reference mutational databases and with an unknown frequency in gnomAD and in other population databases. However, looking to the ADPKD database (https://pkdb.mayo.edu/welcome), we identified an entry with an interpretation as “pathogenic” performed by the database curators according to the study of Audrézet et al. [25]. In the cited paper, authors identified the *PKD2 c.1717-2A>G* variant in two ADPKD families in a comprehensive screening study of a large cohort of affected subjects [25]. Unfortunately, no experimental data are available regarding the effect of the *PKD2* variant on the splicing process. According to the findings of Audrézet et al., the alteration was annotated in the ADPKD variant database as pathogenic with the predicted frameshift effect p.(Leu573fs). Figure 1 shows the damaging output scores from the bioinformatics prediction of pathogenicity of dbscSNV and spliceAI tools. Additionally, the MPA tool calculated a score of 10 associated with the highest splice impact of the variant, while MaxEntScan 3’ss variation resulted in −58.02% and a predicted loss of an acceptor splice-site.

### 3.2. cDNA Analysis

**Figure 2:**
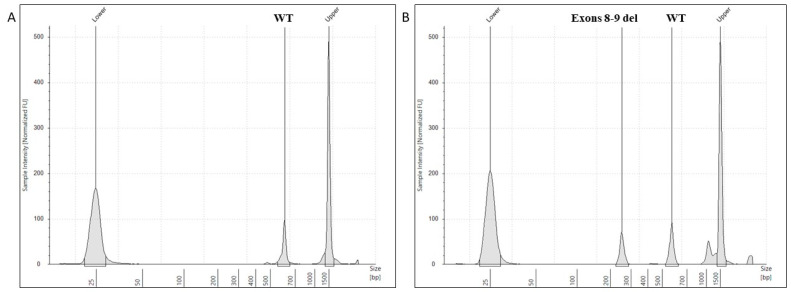
Electrophoretic profiles of cDNA PCR products. This figure shows the electrophoretic profiles obtained from the targeted amplification of cDNA from a control (single wild-type peak, (**A**)) and patient (**B**). The two peaks detected in the analysis of the patient’s cDNA indicated the presence of a wild-type transcript and a mutated transcript (lacking exons 8 and 9), as an effect of the *PKD2 c.1717-2A>G* variant.

From the analysis of cDNA amplification products, we obtained the electrophoretic profiles reported in Figure 2. In Figure 2A, the control sample showed a single fragment with an expected size of approximately 529 bp (the same result was obtained from all controls). By contrast, the PCR products obtained from the patient consisted of two fragments, one corresponding to the wild-type allele of approximately 529 bp and one of 255 bp (Figure 2B).

**Figure 3:**
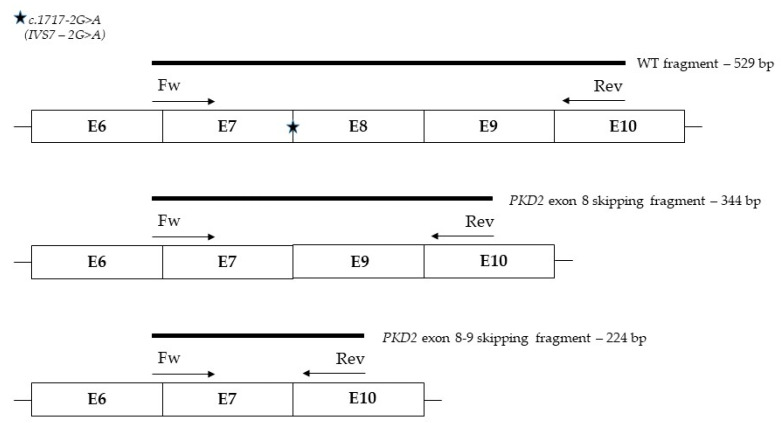
Graphical representation of the PCR strategy adopted. This figure shows the schematic representation of the *PKD2* gene region involved in the splicing event and the expected PCR products, according to primer design. The wild-type, exon 8 skipping, and exon 8 and 9 skipping fragment lengths are reported.

Surprisingly, the latter PCR fragment was smaller than the expected fragment including the sole putative exon 8 skipping (i.e., 344 bp) (Figure 3).

**Figure 4:**
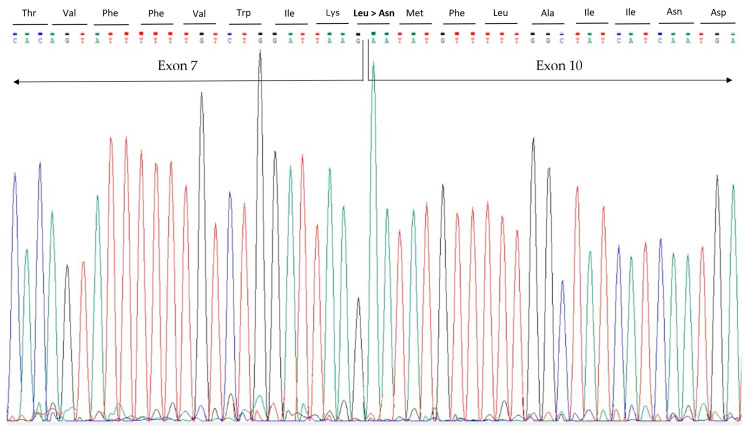
Sanger sequencing of cDNA. This figure shows the Sanger sequencing analysis of the 255 bp PCR product with the merging of exons 7 and 10. The rearrangement causes the amino acidic substitution p.Leu573Asn.

Then, the smaller product was removed from the gel and sequenced, revealing the simultaneous skipping of both exons 8 and 9 of the *PKD2* gene, with a retained reading frame and a merging of exons 7–10. This event causes the loss of 100 amino acids (Figure 4).

## 4. Discussion

RNA splicing is a key post-transcriptional regulation process of gene expression and allows the formation of mature messenger RNA (mRNA) from a precursor messenger RNA (pre-mRNA) transcript [26]. In the molecular evaluation path of a patient affected by ADPKD, we identified the single-base substitution *c.1717-2A>G* involving the acceptor splice site within intron 7 of the *PKD2* gene, causing the simultaneous skipping of the two downstream exons 8 and 9. This outcome could be believed as an uncommon finding, considering that variants involving canonical splice sites or adjacent regions generally lead to the alteration of the splicing via single-exon skipping, short deletions, or insertions in the mature mRNA [27,28]. To date, few examples of double-exon skipping, related to the occurrence of a single-base substitution, have been reported in the literature [29,30,31,32,33,34].

We speculated that the molecular basis of the observed effect of the *c.1717-2A>G* variant on the *PKD2* transcript could follow the splicing model described by Takahara et al. [34]. In this study, the authors reported that the order of intron removal influences the splicing outcome, describing a mutation in the splicing acceptor site of intron 4 of the *COL5A1* gene that led to a simultaneous skip of exons 5 and 6. Moreover, the aberrant splicing outcome differs depending on the splicing site type involved by the substitution (i.e., acceptor or donor) [34].

In our case, the *PKD2* variant *c.1717-2A>G* involves the 5′ acceptor splice site of intron 7, and we can assume that the order of removal of introns 7-8-9 can explain the dual-exon skipping. IVS8 (1715 bp) is smaller than IVS7 (3927 bp) and IVS9 (3802 bp), and, according to the Takahara model, it could be rapidly removed in the mRNA processing with respect to introns 7 and 9. This would result in the fusion of exons 8 and 9 that is successively skipped as a “single-exon” as an effect of the *PKD2 c.1717-2A>G* variant.

From the RT-PCR analysis, we obtained a single aberrant product that seems to be the major outcome of the altered *PKD2* splicing pathway. However, we cannot rule out the hypothesis that other alternative splicing products, subjected to nonsense-mediated decay, can also be formed as an effect of the *c.1717-2A>G* mutation. Probably, the loss of the second exon observed in our patient recreates an in-frame product that is the most abundant but likely producing a non-functional protein. The *PDK2* gene (4q21-q22) consists of 15 exons (NM_000297.3) encoding for the integral membrane PC2 protein, a member of the 6-transmembrane spanning transient receptor potential (TRP) ion channel family. Particularly, PC2 is characterised by six transmembrane domains (S1-S6) and intracellular N- and C-terminal regions. Exons 8 and 9 of the *PDK2* gene are predicted to code for a transmembrane core region of the channel (residues 573–673) and the lack of this region affects the channel structure and function [35,36,37,38].

In Table 1, we collected the *PKD2* splice-site variants currently reported in the main mutational databases and literature. Splicing alterations spread throughout the gene from IVS1 to IVS14, without hot spots or clustering regions. However, fewer studies have investigated splicing effects caused by intronic/exonic variants as a pathogenesis of ADPKD [39] and, to the best of our knowledge, this is the first evidence of a dual-exon skipping event in the *PKD2* gene.

## 5. Conclusions

ADPKD is a common and severe clinical condition leading to kidney failure and cardiovascular disease in most affected individuals. Genetic evaluation has taken on an increasingly central role in the diagnosis and in the familiar screening and monitoring. Here, we have performed a comprehensive analysis of a rare *PKD2* variant involving the acceptor splice site of intron 7, reporting for the first time novel evidence related to the alternative and rare splicing mechanisms in this gene. This study emphasises the significance of assessing the effect of SNVs at the mRNA level in ADPKD by adding new insights to the pathogenesis of the disease.

## Figures and Tables

**Table 1:** Splice site variants of PKD2 gene reported in the literature and in the main mutational databases.

Region	cDNA Reference Sequence *	Reference	Mutational Database and Annotation
IVS1	c.595+2T>C	[40]	ClinVar (ID1068903—P)
	c.595+1G>A	[39]	ClinVar (ID1072849—P)
	c.595+1G>C	[25]	ClinVar (ID811939—P)
	c.596-12_599del	[9]	ClinVar (ID2506174—P)
	c.596-3A>G	[25]	N/A
	c.596-3_596-2insTGS	N/A	LOVD (ID0000886426—LP)
	c.596-1G>T	N/A	ClinVar (ID586315—P)
IVS2	c.709+1G>A	[9]	ClinVar (ID92797—P)
	c.709+1G>T	N/A	ClinVar (ID972824—P)
	c.710-2A>G	[9]	ClinVar (ID1285124—P)
	c.710-1G>A	N/A	ClinVar (ID1255534—P)
EX3	c.843G>A	N/A	ADPKD (LP); ClinVar (ID1255690—VUS)
IVS3	c.843+1G>A	[25]	ClinVar (ID997217—P)
IVS3	c.843+1G>T	N/A	LOVD (ID0000089511—P)
IVS3	c.843+2T>C	N/A	ADPKD (P)
IVS3	c.843+3A>G	N/A	ADPKD (P)
IVS3	c.844-2A>G	N/A	ClinVar (ID997239—P)
IVS4	c.1094+1del	N/A	ClinVar (ID1699919—LP)
IVS4	c.1094+1G>A	[41]	ClinVar (ID280008—P)
IVS4	c.1094+1G>C	[42]	ClinVar (ID 997171—P)
IVS4	c.1094+2T>G	[39]	ADPKD (P)
IVS4	c.1094+3_+6del	[39]	ClinVar (ID434014—P)
IVS4	c.1095-2A>G	[25]	ClinVar (ID1916255—P)
IVS4	c.1095-1G>T	N/A	ClinVar (ID2445831—LP)
IVS4	c.1095-5A>G	[40]	ADPKD (P)
IVS5	c.1319+1G>A	[43]	ClinVar (ID430967—P)
IVS5	c.1319+1G>T	[40]	ADPKD (P)
IVS5	c.1320-2del	N/A	ClinVar (ID638001—P)
IVS5	c.1320-1G>A	[25]	ADPKD (P)
EX6	c.1480G>T	[9,38]	ADPKD (P)
IVS6	c.1548+1G>A	N/A	ClinVar (ID562283—P)
IVS6	c.1549-1G>A	N/A	ClinVar (ID972872—P)
EX7	c.1716G>A	[44]	ADPKD (P)
IVS7	c.1716+1T>A	[25]	ADPKD (P)
IVS7	c.1716+2T>A	[45]	ClinVar (ID997114—P)
IVS7	c.1717-3C>G	[9]	ADPKD (P)
IVS7	c.1717-2A>G	[25]; this study	ADPKD (P)
IVS7	c.1717-1G>A	[46]	ADPKD (P)
IVS8	c.1898+5G>A	[40,46]	ClinVar (ID448033—P)
IVS8	c.1898+1G>A	[40]	ADPKD (P)
IVS8	c.1899-2A>T	N/A	ClinVar (ID2443217—P)
EX9	c.2019G>A	N/A	ADPKD (P)
IVS9	c.2019+1G>A	N/A	ClinVar (ID477626—P)
IVS9	c.2019+2T>A	[22]	ADPKD (P)
IVS9	c.2019+1_2019+5del	N/A	ClinVar (ID829998—P)
IVS9	c.2020-2A>G	N/A	ClinVar (ID1973472—LP)
IVS9	c.2020-2_-1del	[42,47]	ADPKD (P)
IVS9	c.2020-2del	N/A	ClinVar (ID1328420—LP)
IVS9-EX10	c.2020-1_2020del	N/A	ClinVar (ID449307—LP)
IVS10	c.2118-2A>G	N/A	ADPKD (P)
IVS10	c.2118+1G>C	N/A	ClinVar (ID976823—P)
IVS10	c.2119-2A>G	N/A	ClinVar (ID974544—P)
IVS11	c.2240+1G>A	[45]	ClinVar (ID448034—P)
IVS11	c.2240+1G>T	[41]	ADPKD (P)
IVS11	c.2240+1G>C	N/A	ClinVar (ID872745—LP)
IVS11	c.2241-2A>G	[9]	ClinVar (ID562263—P)
IVS11	c.2241-1G>T	[40]	ADPKD (P)
IVS12	c.2358+1G>A	[40]	ClinVar (ID2664070—P)
IVS12	c.2358+1G>T	N/A	ClinVar (ID 2428817—P)
IVS12	c.2358+1G>C	N/A	ClinVar (ID1693454—P)
IVS13	c.2522+1_2522+2del	N/A	ClinVar (ID 975054—P)
IVS13	c.2522+1G>T	N/A	ClinVar (ID1806275—P)
IVS13	c.2523-1G>A	[48]	ClinVar (ID440150—P)
IVS14	c.2670+1G>A	N/A	LOVD (ID0000089601—P)
IVS14	c.2671-2A>G	[40]	ADPKD (P)

* *PKD2* gene reference transcript NM_000297.3. N/A: Not Available; P: Pathogenic; LP: Likely Pathogenic. ClinVar (https://www.ncbi.nlm.nih.gov/clinvar/, accessed on 15 June 2024); ADPKD Variant Database (https://pkdb.mayo.edu/welcome).

## Data Availability

Data are available from the authors upon request.
